# High basal NF-*κ*B activity in nonpigmented melanoma cells is associated with an enhanced sensitivity to vitamin D3 derivatives

**DOI:** 10.1038/bjc.2011.458

**Published:** 2011-11-17

**Authors:** Z Janjetovic, A A Brozyna, R C Tuckey, T-K Kim, M N Nguyen, W Jozwicki, S R Pfeffer, L M Pfeffer, A T Slominski

**Affiliations:** 1 Department of Pathology and Laboratory Medicine, Center for Cancer Research, Department of Medicine, University of Tennessee Health Science Center, 930 Madison Avenue, RM525, Memphis, TN, 38163, USA; 2 Department of Tumor Pathology and Pathomorphology, Oncology Centre – Prof. Franciszek Łukaszczyk Memorial Hospital, Collegium Medicum, Nicolaus Copernicus University, 85-067, Bydgoszcz, Poland; 3 School of Biomedical, Biomolecular and Chemical Sciences, University of Western Australia, Crawley, Western Australia 6009, Australia

**Keywords:** melanoma, melanin pigmentation, NF-*κ*B, vitamin D_3_

## Abstract

**Background:**

Melanoma is highly resistant to current modalities of therapy, with the extent of pigmentation playing an important role in therapeutic resistance. Nuclear factor-*κ*B (NF-*κ*B) is constitutively activated in melanoma and can serve as a molecular target for cancer therapy and steroid/secosteroid action.

**Methods:**

Cultured melanoma cells were used for mechanistic studies on NF-*κ*B activity, utilising immunofluorescence, western blotting, EMSA, ELISA, gene reporter, and estimated DNA synthesis assays. Formalin-fixed, paraffin-embedded specimens from melanoma patients were used for immunocytochemical analysis of NF-*κ*B activity *in situ*.

**Results:**

Novel 20-hydroxyvitamin (20(OH)D_3_) and classical 1*α*,25-dihydroxyvitamin D_3_ (1,25(OH)_2_D_3_) secosteroids inhibited melanoma cell proliferation. Active forms of vitamin D were found to inhibit NF-*κ*B activity in nonpigmented cells, while having no effect on pigmented cells. Treatment of nonpigmented cells with vitamin D3 derivatives inhibited NF-*κ*B DNA binding and NF-*κ*B-dependent reporter assays, as well as inhibited the nuclear translocation of the p65 NF-*κ*B subunit and its accumulation in the cytoplasm. Moreover, analysis of biopsies of melanoma patients showed that nonpigmented and slightly pigmented melanomas displayed higher nuclear NF-*κ*B p65 expression than highly pigmented melanomas.

**Conclusion:**

Classical 1,25(OH)_2_D_3_ and novel 20(OH)D_3_ hydroxyderivatives of vitamin D3 can target NF-*κ*B and regulate melanoma progression in nonpigmented melanoma cells. Melanin pigmentation is associated with the resistance of melanomas to 20(OH)D_3_ and 1,25(OH)_2_D_3_ treatment.

Melanoma is a neoplasm of melanocytic origin with an environmental aetiology that is linked to overexposure to solar radiation. Melanoma incidence depends on race and ethnicity, indicating that melanin may play a protective role (Slominski *et al*, 2004; Lin and Fisher, 2007). Skin exposure to ultraviolet B (UVB) radiation generates vitamin D_3_, a crucial hormone/prohormone, through photochemical transformation of 7-dehydrocholesterol (Holick, 2003). The sequential hydroxylation of vitamin D3 at positions 25 and 1 produces the active form, 1*α*,25-dihydroxyvitamin D_3_ (1,25(OH)_2_D_3_; calcitriol), which regulates calcium body homeostasis and has immunomodulatory, antiproliferative, and anticancer activity (Holick, 2003; Deeb *et al*, 2007; Bikle, 2010). These pleiotropic activities are regulated by interaction with the vitamin D nuclear receptor (VDR) (Holick, 2003; Bikle, 2010). The novel vitamin D_3_ hydroxyderivative (20-hydroxyvitamin (20(OH)D_3_)), a product of cytochrome *P*450scc action (Slominski *et al*, 2005), affects the proliferation and differentiation of human keratinocytes (Zbytek *et al*, 2008) and leukaemic cells (Slominski *et al*, 2010), but also inhibits nuclear factor-*κ*B (NF*κ*B) activity in keratinocytes (Janjetovic *et al*, 2009). As 20(OH)D_3_ is noncalcemic (Slominski *et al*, 2010), it has a significant therapeutic potential for treating hyperproliferative disorders. Melanoma is the most aggressive form of skin cancer and is notoriously resistant to all current modalities of cancer therapy once the metastatic process has begun (Soengas and Lowe, 2003). Melanin pigment, which protects cells against the harmful actions of UV radiation attenuating melanomagenesis (Slominski *et al*, 2004), can paradoxically contribute to the resistance of melanoma to different forms of therapy (Slominski *et al*, 1998; Kinnaert *et al*, 2000; Meyskens *et al*, 2007; Brozyna *et al*, 2008).

Nuclear factor-*κ*B (NF-*κ*B) plays an important role in inflammation and cancer development (Van Waes, 2007), and is constitutively activated in many human cancers, including melanoma (Franco *et al*, 2001; Dolcet *et al*, 2005; Karin, 2006). In mammals, the NF-*κ*B family includes NF-*κ*B1 (p105/p50), NF-*κ*B2 (p100/p52), Rel A (p65), Rel B, and cRel. In most cells, NF-*κ*B is found bound to I*κ*B as an inactive complex in the cytoplasm. Phosphorylation and subsequent degradation of I*κ*B proteins result in NF-*κ*B translocation into the nucleus, where it can bind to specific gene promoters and activate transcription. The activation of NF-*κ*B is mediated through the activation of the I*κ*B kinase (IKK) complex, which catalyses I*κ*B phosphorylation. Nuclear factor-*κ*B is a potential molecular target for cancer therapy and for steroid action (Van Waes, 2007). As increased NF-*κ*B activity appears to play an important role in maintaining the aggressive nature of melanoma (Yang and Richmond, 2001), and melanin pigment affects the sensitivity of melanoma cells to chemotherapy (Slominski *et al*, 2004), we analysed the relationship between pigmentation and NF-*κ*B activity in a well-defined cell culture model of inducible melanin pigmentation (Brozyna *et al*, 2008; Slominski *et al*, 2009). This relationship was also validated in clinical biopsies from melanoma patients. Furthermore, as active forms of vitamin D inhibit NF-*κ*B (Riis *et al*, 2004; Janjetovic *et al*, 2009, 2010), we examined whether pigmentation affects the antiproliferative activity of novel (20(OH)D_3_) and classical (1,25(OH)_2_D_3_) forms of vitamin D_3_ and defined the mechanism of their action on NF-*κ*B.

## Materials and methods

### Cell culture

Human SKMEL-188 melanoma cells (a kind gift from Dr Ashok Chakraborty, Yale University, New Haven, CT, USA), established from a human metastatic melanoma, were maintained in Ham's F-10 medium deficient in melanin precursor L-tyrosine (∼10 *μ*M concentration; Cellgro, Manassas, VA, USA) and supplemented with glucose, L-glutamine, pyridoxine hydrochloride (Cellgro), 5% fetal bovine serum (FBS) (Sigma, St Louis, MO, USA), and 1% penicillin/streptomycin/amphotericin antibiotic solution (Sigma). Melanin pigmentation was induced by changing the media to 25 : 75 mixture of F-10 and Dulbecco's modified Eagle's medium (DMEM; Cellgro) containing increased concentration of L-tyrosine (∼420 *μ*M), supplemented with 5% FBS (Slominski *et al*, 1999). During treatment with vitamin D derivatives, 5% charcoal/dextran-treated bovine serum (HyClone, Logan, UT, USA) was used. The 20(OH)D_3_ was synthesised enzymatically by the action of bovine cytochrome *P*450scc on vitamin D and purified by TLC and HPLC as described (Slominski *et al*, 2005).

### Cell proliferation assay

Melanoma cells were seeded in a 24-well plate (TPP, Trasadingen, Switzerland) and grown until reaching ∼80% confluence. Cells were serum starved overnight and then treated for 24 or 48 h with 0.1–100 nM 1,25(OH)_2_D3 or 20(OH)D3 that was diluted in medium containing charcoal-stripped serum followed by the addition of 1 *μ*Ci ml^–1^ [^3^H]-thymidine (Moravek Biochemicals Inc., Brea, CA, USA) to the concentration of 1 *μ*Ci ml^–1^ for 4 h. The excess unbound thymidine was removed by washing with 1 × PBS. Cells were washed with 1 × PBS and subjected to precipitation with 10% trichloroacetic acid (TCA) (Sigma, Munich, Germany) and then dissolved with 1 N NaOH. The precipitate was collected in vials and thymidine incorporation was determined in liquid scintillation counter (Beckman LS 6000, Santa Clara, CA, USA).

### Gene reporter assays

Cells were co-transfected with the NF*κ*B-Luc construct and a *Renilla* luciferase reporter gene (Promega, Madison, WI, USA), which served as normalisation control, using Lipofectamine Plus (Invitrogen, Carlsbad, CA, USA) in F-10 medium, as described previously (Zbytek *et al*, 2008). Cells were also transfected with p-Luc (Promega) that served as a vector control. At 24 h after transfection, cells were incubated with fresh medium containing 100 nM 1,25(OH)_2_D_3_, 20(OH)D_3_, or ethanol (as vehicle) for 1, 4 8, 16, 24, or 48 h. Firefly luciferase and *Renilla* luciferase activity was measured on a TD-20/20 luminometer (Turner Designs, Sunnyvale, CA, USA) following the protocol for DLR Assay (Promega). The resulting promoter-specific firefly activity was normalised to the *Renilla* signal and expressed relative to the activity obtained in control (untreated) cells.

### Preparation of cell extracts

Nuclear extracts were prepared as previously described using a cell extraction kit (Active Motif, Carlsbad, CA, USA). Cells were harvested and resuspended in 1 × hypotonic buffer. After incubation for 15 min on ice, the detergent was added and the suspension was centrifuged for 30 s at 14 000 **g** (Du *et al*, 2009). Supernatant was collected as cytoplasmic extract. The cell pellet was further resuspended in complete lysis buffer with DTT and protease inhibitor cocktail (Sigma). After 30 min of incubation on ice with shaking, the cell extract was centrifuged (14 000 **g** for 10 min at 4 °C). The resultant supernatant was considered the nuclear extract. Protein concentration was determined using a BCA protein assay kit (Thermo Scientific, Pierce, Rockford, IL, USA).

Whole-cell extracts were prepared by lysing cells in complete lysis buffer with DTT and protease inhibitor cocktail. After incubation for 60 min on ice, the suspension representing the whole-cell extract was centrifuged (13 226 **g**) for 20 min at 4 °C. The amount of protein was determined using a Bradford protein assay kit (Bio-Rad, Hercules, CA, USA).

### Electrophoretic mobility shift assay (EMSA)

The NF-*κ*B DNA-binding activity was determined by EMSA. Nuclear extract (20 *μ*g) was incubated at 22 °C for 30 min with NF-*κ*B ^32^P-labelled oligonucleotide and gel shift binding buffer consisting of 2.5 mmol l^–1^ DTT, 0.25% Tween-20, and 0.25 mg ml^–1^ poly(dI):poly(dC). For supershift assays, 1 *μ*g antibody against p50 and p65 (NCI Preclinical Repository, Rockville, MD, USA) or VDR (Santa Cruz Inc., Santa Cruz, CA, USA) were added to the nuclear extract before DNA binding and incubated for 20 min at 22 °C. Loading dye (2 *μ*l of 10 × ) was added to each sample and samples were separated on 5% TBE gels at 80 V for 3 h. The gel was dried and the bands were quantified by phosphoimage autoradiography (Cyclone, Packard, Palo Alto, CA, USA).

### Western blotting analysis

Using western blotting, NF-*κ*B p65, lamin A, and *β*-actin levels were assessed, as described previously (Janjetovic *et al*, 2009). Primary antibodies used were rabbit polyclonal antibody against p65 (Santa Cruz Inc.; 1 : 500 dilution), lamin A (Santa Cruz Inc.; 1 : 200 dilution), and *β* actin-peroxidase (Sigma; 1 : 7000 dilution). The secondary antibody used was anti rabbit-HRP (Santa Cruz Inc.; 1 : 7000 dilution). The immunoblot was developed with the ECL reagent (Pierce, Thermo Scientific, Rockford, IL, USA) and visualised on a Kodak Imager (Rochester, New York, NY, USA).

### Immunoprecipitation analysis

Cells were lysed in RIPA buffer (Cell Signaling, Beverly, MA, USA) on ice for 1 h. After centrifugation (15 339 **g** for 20 min at 4 °C), the lysates were incubated with 25 *μ*l of Protein A/G PLUS-Agarose beads (Santa Cruz Inc.) and 1 *μ*g of normal mouse serum for 3 h with shaking at 4 °C, pelleted by centrifugation for 10 min at 4 °C, and incubated overnight at 4 °C on a rocking platform with 25 *μ*l of Protein A/G PLUS-Agarose beads and 2 *μ*g of anti-VDR antibody (D-6) (Santa Cruz Inc.). Immunoprecipitates were collected by centrifugation, washed four times with RIPA buffer, separated by SDS–PAGE, and subjected to immunoblotting. The primary antibody used for immunoblotting was mouse monoclonal antibody to VDR (Santa Cruz Inc.; 1 : 500 dilution) or the mouse monoclonal antibody to ERp57 (Santa Cruz Inc.; 1 : 50 000 dilution). The secondary antibody used was TrueBlot ULTRA HRP anti-mouse IgG (eBioscience, San Diego, CA, USA; 1 : 1000 dilution). The immunoblots were developed with the ECL reagent (Pierce, Thermo Scientific) and visualised on a Kodak Imager.

### Enzyme-linked immunosorbent assay

To monitor p65 translocation into the nucleus, SKMEL-188 cells were grown to 80% confluency and then treated with 100 nM 1,25(OH)_2_D_3_ or 20(OH)D_3_ for 0, 0.5, 1, 4, or 24 h. The cells were harvested, nuclear and cytoplasmic extracts prepared, and nuclear p65 levels were determined using an enzyme-linked immunosorbent assay (ELISA) according to the manufacturer's protocol (IMGENEX, San Diego, CA, USA). The amount of nuclear and cytoplasmic p65 was detected by adding a secondary antibody followed by alkaline-phosphatase-conjugated antibody. The absorbance value for each well was determined at 405 nm using a microplate reader.

### Immunohistochemical analysis

Samples of melanoma were obtained from 79 patients (35 females and 44 males, age range from 25 to 90 years, median 59.8±14.3) treated at the Oncology Centre – Prof. Franciszek Łukaszczyk Memorial Hospital, Bydgoszcz, Poland. Melanomas with different grades of pigmentation (amelanotic (*n*=27), moderately (*n*=32), and strongly pigmented (*n*=19)) were classified according to Clark's stage and Breslow's depth. Samples included 16 melanomas at Clark's stage I, 7 at Clark's stage II, 22 at Clark's stage III, 27 at Clark's stage IV, and 13 at Clark's stage V. Breslow's thickness of studied melanomas was 0–1 mm, 17 cases; 1.1–2.0 mm, 10 cases; 2.1–3.0 mm, 9 cases; 3.1–4.0 mm, 5 cases; and >4.1 mm, 32 cases. Melanin content was graded as 0 if melanin was absent, 1 if melanin was visible in up to 50% of cells, and 2 if melanin was present in >50% of cells. Sections were made as previously described with minor modifications (Janjetovic *et al*, 2009). In brief, formalin-fixed, paraffin-embedded sections were dewaxed with xylene and gradually hydrated. After antigen retrieval by heating in Tris/EDTA (pH 9.0), sections were blocked with 2% BSA and incubated overnight at 4 °C with anti-p65 antibody (Santa Cruz Biotechnology; dilution 1 : 150 in antibody diluent) (Dako, Glostrup, Denmark). AlexaFluor 488-conjugated secondary anti-rabbit antibody (dilution 1 : 500 in TBS) was added for 60 min (Invitrogen), cells were washed with PBS, and mounted in Vectashield mounting medium containing propidium iodide (PI) (Vector Laboratories, Burlingame, CA, USA) for nuclear visualisation. Primary antibody was omitted in negative controls. Consecutive sections were stained with H&E. Sections were viewed at × 40 magnification with a BX-50 epifluorescence microscope (Olympus, Tokyo, Japan) equipped with FITC and TRITC filters and photo-documented using a Nikon DS (Melville, NY, USA) digital camera. The study was approved by the Committee of Ethics of Scientific Research of Collegium Medicum of Nicolaus Copernicus University, Poland. Sections were evaluated for the intensity, extent, and localisation of staining for the p65 subunit of NF-*κ*B within the melanoma cells (Li *et al*, 2010).

The proliferation activity of melanomas was assessed by Ki-67 immunocytochemistry. Deparaffinisation and antigen retrieval was achieved by boiling sections in high pH buffer in PT Link module (Dako). After quenching of the endogenous peroxidase activity with 3% H_2_O_2_, sections were incubated with anti-Ki67 primary monoclonal mouse antibody, clone MIB-1 (Dako; dilution 1 : 100) with EnVision FLEX Antibody Diluent (Dako) for 30 min at room temperature. Next, HRP-labelled secondary, anti-mouse antibody (EnVision+System HRP Labelled Polymer Anti-Mouse; Dako) was added at room temperature for 30 min, followed by incubation with DAB (Dako) for 5 min. Then, sections were counterstained with haematoxylin, dehydrated, and mounted (Thermo Shandon Ltd, Rochester, New York, NY, USA/Thermo Fisher Scientific Inc., Kalamazoo, MI, USA). The positive controls for Ki67 staining were sections of lymph node. Ki67-labelled sections were evaluated under Nikon Eclipse 80i light microscope. The Ki67 expression was evaluated as percentage of Ki67-positive melanoma cells. Moreover, mitotic index was estimated and defined as number of cells in mitosis per 1 mm^2^. The VDR immunostaining was performed as previously described (Brozyna *et al*, 2011).

### Immunofluorescence staining

The SKMEL-188 cells were seeded onto cover glasses in six-well plates and treated with 100 nM 20(OH)D_3_, 1,25(OH)_2_D_3_, or vehicle (0.1%. ethanol) for 1 h. Following treatment, cells were washed with PBS, fixed in 4% paraformaldehyde, permeabilised in 0.2% Triton X-100 in PBS for 5 min, and washed with PBS. After blocking in 2% BSA for 30 min, primary antibody (NF*κ*B-p65 (1 : 100) in 1% BSA) was added and incubated overnight at 4 °C. Cells were washed with PBS and secondary antibody (Alexa Fluor 488 (1 : 500 dilution in PBS), Invitrogen) was applied for 1 h in the dark. After washing and mounting with Vectashield mounting medium containing PI (Vector Laboratories), cells were examined with a fluorescent microscope and photographed at × 40 magnification (Janjetovic *et al*, 2009).

### VDR translocation

To determine VDR translocation from cytoplasm to the nucleus induced by 20(OH)D_3_, SKMEL-188 cells were transduced with pLenti-CMV-VDR-EGFP-pgk-puro to express VDR and EGFP fusion protein (Slominski *et al*, 2011). The cells were incubated with hydroxyvitamin D_3_ derivatives for 2 h, followed by fixation with 4% paraformaldehyde and mounting with fluorescence mounting medium, and were analysed on a fluorescent microscope. Data are presented as a percentage of cells with fluorescent nuclei relative to the total cell number.

### Statistical analysis

Data are presented as mean±s.d. and analysed using Student's *t*-test (for two groups) and appropriate *post hoc* test (for more than two groups) using Prism 4.00 (GraphPad Software, San Diego, CA, USA). Statistically significant differences are denoted as ^*^P<0.05, ^**^P<0.01, and ^***^P<0.001.

## Results

### Characterisation of NF-*κ*B activity in nonpigmented *vs* pigmented melanoma cells

In previous studies (Janjetovic *et al*, 2009, 2010), we established that vitamin D derivatives target the NF-*κ*B pathway in human keratinocytes. In this study, we examined the role of active forms of vitamin D in regulation of NF-*κ*B activity in melanoma cells that depended on melanin pigmentation. For *in vitro* studies we used the human SKMEL-188 melanoma line that is amelanotic when cultured in F-10 medium relatively deficient in melanin precursors, but produces melanin pigment when cultured in a mixture of DMEM and F-10 (75 : 25) that contains an increased concentration of melanin precursors including L-tyrosine (Slominski *et al*, 1999). The medium rich in L-tyrosine induces melanin pigmentation and changes responsiveness of melanoma cells to different treatment protocols (Brozyna *et al*, 2008; Slominski *et al*, 2009).

**Figure 1:**
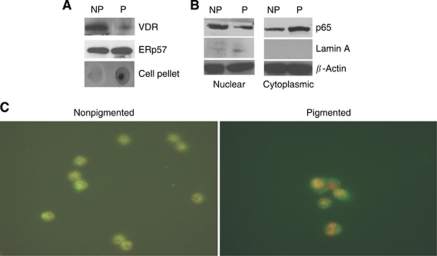
Characterisation of NF-*κ*B and VDR protein in pigmented and nonpigmented melanoma cells. (**A**) Immunoblotting for VDR expression and ERp57 of extracts of nonpigmented (NP) and pigmented cells (P) and picture of pigmented and nonpigmented cell pellets. (**B**) Immunoblotting of nuclear and cytoplasmic extracts from pigmented (P) and nonpigmented (NP) SKMel-188 cells for the p65 subunit of NF-*κ*B revealed its nuclear localisation in nonpigmented cells, whereas p65 was predominantly found in the cytoplasm of pigmented cells. Extracts were subjected to immunoblotting with NF-*κ*B p65, lamin A, and *β*-actin. (**C**) Immunofluorescence staining of p65 revealed its nuclear location in nonpigmented cells, whereas in pigmented cells p65 was localised predominantly in cytoplasm.

First, we examined whether the induction of melanin pigmentation can affect the expression of the vitamin D receptor. Immunoprecipitation and subsequent western blot analysis (Figure 1A) reveals that pigmented SKMEL-188 cells express less VDR protein than nonpigmented cells. In addition, blotting for anti-ERp57, which may represent a putative cell membrane vitamin D receptor (Khanal and Nemere, 2007), showed that the ERp57 protein was equally expressed in pigmented and nonpigmented cells. Therefore, melanin content is inversely proportional to VDR protein expression.

In general, the most common form of NF-*κ*B in activated cells is a nuclear complex of the p50 and p65 subunits. To examine the subcellular localisation of the p65 NF-*κ*B subunit in SKMEL-188 melanoma cells, we prepared cytoplasmic and nuclear extracts from pigmented and nonpigmented melanoma cells. Lysates from equivalent numbers of cells were analysed by western blotting with anti-p65. Results revealed that higher nuclear levels of p65 were present in nonpigmented SKMEL-188 melanoma cells than in pigmented cells. Conversely, higher cytoplasmic levels of p65 were observed in pigmented cells than in nonpigmented cells (Figure 1B). To further characterise the activation of NF-*κ*B in melanoma cells, we performed immunofluorescent staining for p65 in pigmented and nonpigmented SKMEL-188 cells. As shown in Figure 1C, prominent p65 nuclear staining was found in nonpigmented melanoma cells, whereas p65 was mainly localised in the cytoplasm of pigmented cells.

### 20(OH)D_3_ reduces NF-*κ*B DNA activation in melanoma cells

**Figure 2:**
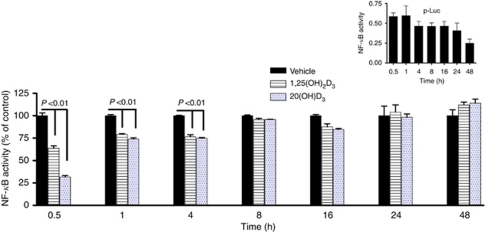
The effects of treatment with 20(OH)D_3_ or 1,25(OH)_2_D_3_ on NF-*κ*B-dependent transcriptional activity in nonpigmented melanoma cells. Cells were transiently co-transfected with NF-*κ*B-Luc reporter plasmid and *Renilla* reporter for 24 h, and then treated with 100 nM 20(OH)D_3_ or 1,25(OH)_2_D_3_, or vehicle (ethanol) for the indicated times. Luciferase activity was measured in four independent experiments and data are presented as percentile of control (mean±s.d.). Transfection with p-Luc served as negative control (insert). Data are presented as a percent (%) of control.

To determine whether 20(OH)D_3_ or 1,25(OH)_2_D_3_ affected NF-*κ*B-driven transcriptional activity, pigmented and nonpigmented melanoma cells were transfected with a NF-*κ*B-dependent luciferase reporter construct (NF-*κ*B-p-Luc). The experiments revealed that constitutive NF-*κ*B-driven transcriptional activity in nonpigmented melanoma cells was reduced by both secosteroids in a time-dependent manner (Figure 2). Treatment for 30 min with 1,25(OH)_2_D_3_ and 20(OH)D_3_ resulted in 50% and 80% reduction in NF-*κ*B-dependent luciferase activity, respectively. After 1 and 4 h, the inhibitory effect was less prominent (∼20% reduction), and after 8 h of treatment, NF-*κ*B-dependent luciferase activity returned to baseline levels. In contrast, treatment of melanised melanoma with either 20(OH)D_3_ or 1,25(OH)_2_D_3_ was without any effect on NF-*κ*B-driven transcriptional activity (data not shown).

**Figure 3:**
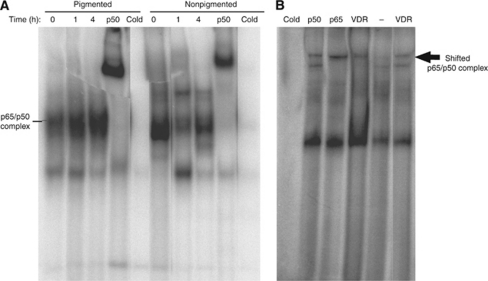
The DNA-binding activity of NF-*κ*B in melanoma cells after treatment with 20(OH)D_3_. (**A**) Nuclear extracts were prepared from nonpigmented and pigmented melanoma cells alone or treated with 100 nM 20(OH)D_3_ for indicated time periods, and were subjected to EMSA. Cold represents extracts preincubated with a 50-fold excess of unlabelled oligonucleotide. (**B**) Nuclear extracts from cells after 1 h of exposure to the vitamin D3 derivative were subjected to supershifts with anti-p50, p65, VDR (1 : 10 dilution, lane 4 and 1 : 5 dilution, lane 6), or with no addition of antibody (lane 5).

Transcriptional activity of NF-*κ*B is usually associated with an increased DNA binding of NF-*κ*B as determined by EMSA using a consensus NF-*κ*B oligonucleotide probe. As shown in Figure 3A, high constitutive activation of NF-*κ*B DNA-binding activity was observed in nuclear extracts prepared from nonpigmented SKMEL-188 melanoma cells, as compared with extracts prepared from pigmented cells. Treatment of nonpigmented cells with 20(OH)D_3_ resulted in a time-dependent inhibition of NF-*κ*B-dependent DNA-binding activity. The presence of the p50 or p65 subunit within the NF-*κ*B complexes was confirmed by supershift assays with the antibodies directed to these NF-*κ*B subunits (Figure 3B). The specificity of NF-*κ*B binding was shown by competition with a 50-fold excess of unlabelled NK-*κ*B oligonucleotide. Also, the presence of VDR within NF-*κ*B complexes was confirmed by supershift assay with anti-VDR antibody (Figure 3B). Treatment of pigmented cells with 20(OH)D_3_ had no effect on NF-*κ*B activation (Figure 3A).

**Figure 4:**
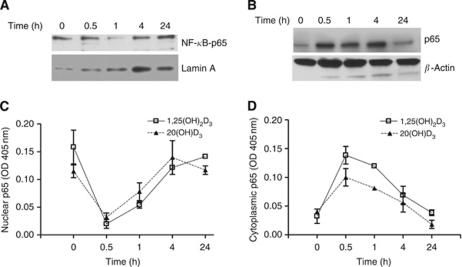
Treatment with 20(OH)D_3_ inhibits nuclear translocation of the p65 NF-*κ*B subunit in nonpigmented melanoma. The cells were treated with 100 nM 20(OH)D_3_ for the indicated time periods. (**A**) Nuclear extracts from cells were subjected to immunoblotting with anti-NF-*κ*B p65, and anti-Lamin A (internal control). (**B**) Cytoplasmic extracts were subjected to immunoblotting with NF-*κ*B p65 and *β*-actin (internal control). The levels of p65 in nuclear (**C**) and cytoplasmic (**D**) extracts were quantified by ELISA using the same amount of proteins (determined by protein assay) and measuring OD at 405 nm. Following the manufacturer's recommendation, p65 standard curve was titrated to determine protein levels.

To further characterise the effects of secosteroids and pigmentation on NF-*κ*B activity, we examined p65 nuclear translocation using a highly sensitive ELISA as well as by western blot and immunofluorescence. Treatment of nonpigmented SKMEL-188 melanoma cells with 20(OH)D_3_ decreased nuclear levels of p65 (Figure 4A) with a subsequent increase in cytoplasmic p65 levels (Figure 4B). Translocation of p65 into the nucleus was greatly inhibited by treatment with 20(OH)D_3_ or 1,25(OH)_2_D_3_ (100 nM for 30 min). By 4 h, the inhibitory effect of the secosteroids on p65 translocation was no longer detected (Figure 4C and D). Treatment of nonpigmented melanoma cells with 20(OH)D_3_ decreased nuclear levels of p65 (Figure 4A) with a subsequent increase in cytoplasmic p65 levels (Figure 4B). In pigmented cells, treatment with 20(OH)D_3_ or 1,25(OH)_2_D_3_ had no effect on the intracellular localisation of p65 (data not shown). These results indicate that 20(OH)D_3_ and 1,25(OH)_2_D_3_ inhibit p65 nuclear translocation in nonpigmented but not in pigmented human melanoma cells. These data were further confirmed by immunofluorescent staining; that is, treatment with 20(OH)D_3_ and 1,25(OH)_2_D_3_ blocked p65 nuclear translocation in SKMEL-188 nonpigmented melanoma cells. Also, as shown in Supplementary Figure 1, secosteroid treatment (100 nM for 1 h) of nonpigmented cells resulted in the retention of p65 in the cytoplasm with little evidence of nuclear staining, whereas p65 nuclear staining was clearly evident in untreated cells. No effect of secosteroid treatment was seen in pigmented melanoma cells (Supplementary Figure 1).

### P65 subunit is mainly localised in the nuclei of nonpigmented melanoma cells

**Figure 5:**
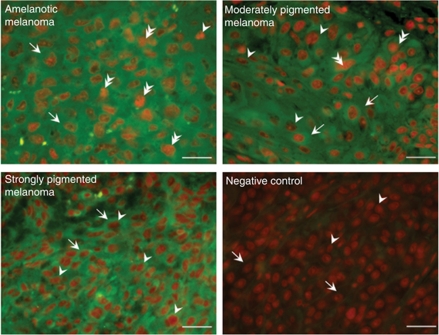
Nuclear factor-*κ*B expression in pigmented and nonpigmented melanoma tissue. Sections of human melanomas were subjected to immunohistochemical staining for the p65 NF-*κ*B subunit. p65 staining with AlexaFluor 488 (green) was merged with nuclei staining with PI (red). Arrows indicate cytoplasm of melanoma cells, arrowheads indicate melanoma cell nuclei, and double arrowheads indicate melanoma cell with p65 localised in nuclei. Scale bars=50 *μ*m.

To further validate our *in vitro* data, we evaluated whether differences in NF-*κ*B activity in human melanoma samples was reflective of their pigmentation. We examined p65 expression by immunocytochemistry of melanomas obtained from 79 patients (35 females and 44 males, age range 25–90 years, median 59.8±14.3). Predominantly nuclear p65 NF-*κ*B staining was observed in 23 out of 27 samples (85.2%) of nonpigmented melanoma, whereas in 18 of 32 samples (56.3%) of moderately pigmented melanomas, p65 was present in both the cytoplasm and nuclei. In contrast, in 15 out of 19 samples (78.9%) of strongly pigmented melanomas, p65 was found exclusively in the cytoplasm (Figure 5). Moreover, the intensity of p65 staining in the cytoplasm was also found to increase with increasing melanoma pigmentation, with a significant difference being observed between nonpigmented (average relative value=1.01) and strongly pigmented melanomas (average relative value=1.37; *P*<0.01). However, the intensity of p65 staining in nuclei decreased with increased pigmentation (0.68 *vs* 0.11 for average relative values, in nonpigmented and strongly pigmented melanomas, respectively). The nuclear staining of p65 was significantly higher (*P*<0.005) in nonpigmented compared with moderately and strongly pigmented melanomas (Table 1). Thus, analysis of the large archival material (79 cases) demonstrated that pigmentation affects NF-*κ*B activity as evidenced by changes in the intracellular distribution of p65 (predominantly nuclear p65 staining in nonpigmented cells as compared with predominantly cytoplasmic p65 staining in pigmented cells). This pattern was comparable with cell culture results showing higher nuclear levels of p65 in nonpigmented than in pigmented melanoma cells (Figure 1). Conversely, higher cytoplasmic levels of p65 were observed in pigmented than in nonpigmented cells (Figure 1).

**Figure 6:**
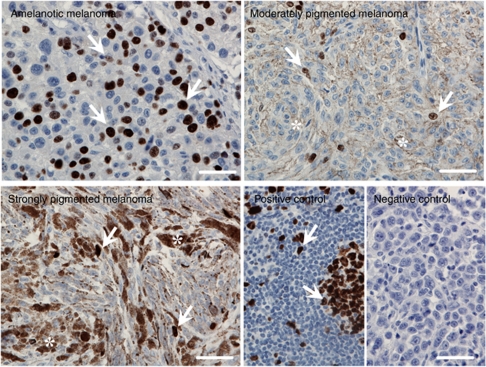
The expression of Ki67 in pigmented and nonpigmented melanoma tissue. Sections of human melanomas were subjected to immunohistochemical staining for Ki67, visualised with DAB and counterstained with haematoxylin. Arrows indicate Ki67-positive cells, and asterisks indicate melanin. Lymph node was used as positive control. Scale bars=50 *μ*m.

**Figure 7:**
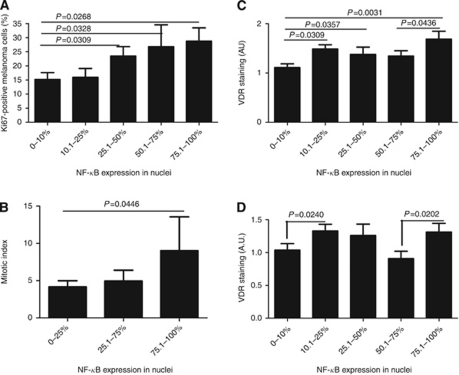
Nuclear factor-κB and VDR expression in melanoma tissue. (**A**) Ki67 expression in melanoma samples with different percentage of NF-*κ*B expression in nuclei. (**B**) The mitotic index of melanoma samples with different percentage of NF-*κ*B expression in nuclei. (**C**) The VDR nuclear immunostaining in melanoma samples with different percentage of NF-*κ*B expression in nuclei. (**D**) The VDR cytoplasmic immunostaining in melanoma samples with different percentage of NF-*κ*B expression in nuclei.

In human melanoma samples, translocation of NF-*κ*B into the cell nuclei was accompanied by a higher proliferation activity as assessed by both Ki67 immunostaining and the cellular mitotic index. Melanomas with <10% of cells exhibiting p65 nuclear staining showed significantly lower proliferation activity assessed by Ki-67 immunohistochemistry (Figure 6) when compared with melanomas with p65 nuclear staining in >25% of cells (Figure 7A). We also observed a correlation between percentage of NF-*κ*B nuclear immunostaining and the percentage of Ki67-positive cells (*r*=0.3144, *P*=0.0034). Similar results were observed when mitotic index was used for growth rate assessment, but statistically significant differences were only observed between melanomas with <25% and >75% of cells with p65 nuclear staining (Figure 7B). However, the percentage of cells with NF-*κ*B nuclear immunostaining also correlated with mitotic index (*r*=0.1977, *P*=0.0492).

**Figure 8:**
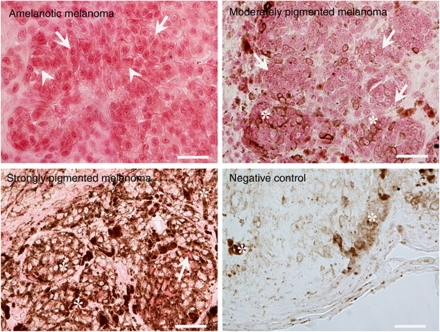
The expression of VDR in pigmented and nonpigmented melanoma tissue. Sections of human melanomas were subjected to immunohistochemical staining for VDR, and visualised with VECTOR Red (alkaline phosphatase substrate). Arrows indicate VDR-positive cell nuclei, arrowheads indicate VDR-positive cytoplasm, and asterisks indicate melanin. Scale bars=50 *μ*m. The color reproduction of this figure is available at the *British Journal of Cancer* online.

The melanoma samples were also assessed for VDR expression by immunohistochemistry (Figure 8). First, we found a decrease of VDR expression in moderately and heavily pigmented melanomas in comparison with amelanotic ones (Figure 8A–C). Second, in melanoma patients, the highest nuclear VDR immunostaining was observed in melanomas with >75% of cells with p65 nuclear staining (Figure 7C), and VDR immunostaining also correlated with the percentage of cells with nuclear NF-*κ*B staining. There was significantly elevated VDR nuclear staining as compared with melanomas with <10% of cells with nuclear NF-*κ*B. The relationship of cytoplasmic VDR immunostaining and translocation of NF-*κ*B into nucleus was less clear, because the lowest cytoplasmic VDR immunostaining was seen in melanomas with <10% and 50.1–75% of cells with nuclear NF-*κ*B (Figure 7D).

### Inhibition of melanoma growth by 20(OH)D_3_ and 1,25(OH)_2_D_3_ is attenuated by melanotic phenotype

**Figure 9:**
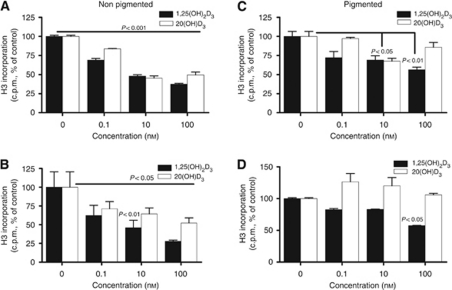
The growth of melanoma cells is inhibited by 20(OH)D_3_ and 1,25(OH)_2_D_3_ . Cells were treated with graded concentrations of 20(OH)D_3_ or 1,25(OH)_2_D_3_ for 24 and 48 h. DNA incorporation of radioactive thymidine was determined. Data are presented as means±s.d. of thymidine incorporation (% of control). Statistical significance was measured using Student's *t*-test. DNA incorporation in nonpigmented cells treated for 24 h (**A**) and 48 h (**B**), and in pigmented cells treated for 24 h (**C**) and 48 h (**D**). Data were obtained from three independent experiments.

To further characterise the effects of secosteroids on melanoma, we examined their effects on SKMEL-188 cell proliferation. After growth in serum-free media for 24 h, nonpigmented and pigmented melanoma cells were treated with varying concentrations of 20(OH)D_3_ or 1,25(OH)_2_D_3_, and cell proliferation rate was determined by thymidine incorporation. As shown in Figure 9, 20(OH)D_3_ and 1,25(OH)_2_D_3_ exhibited a statistically significant inhibitory effect on SKMEL-188 proliferation at 24 h (*P*<0.001; Figure 9A) and 48 h (*P*<0.05) after secosteroid addition (Figure 9B). At the 100 nM concentration, the inhibitory effect on proliferation was significantly greater. Both 20(OH)D_3_ and 1,25(OH)_2_D_3_ had a greater inhibitory effect on the proliferation of nonpigmented melanoma cells as compared with pigmented cells (Figure 9C and D).

**Figure 10:**
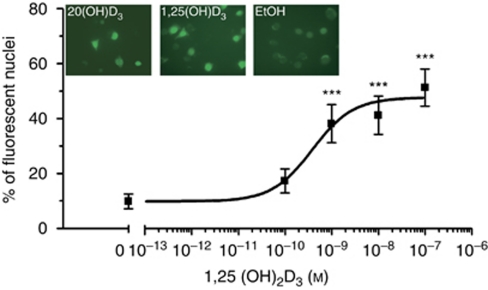
The effects of 1,25(OH)_2_D_3_ and 20(OH)D_3_ on the translocation of VDR from the cytoplasm to the nucleus. Dose-response effect for 1,25(OH)_2_D_3_ is shown with representative VDR translocation induced by 20(OH)D_3_, 1,25(OH)_2_D_3_, and ethanol (vehicle control). The top panel shows the effect of vitamin D3 treatment on cells after 2 h of treatment at the concentration of 100 nM. Data are presented as means±s.d. (*n*⩾6 measurements for one experiment). The differences between control and treatment were analysed using Student's *t*-test: *P*<0.001 (^***^).

Using SKMEL-188 stably expressing VDR and GFP fusion protein (Slominski *et al*, 2011), we also examined the effect of vitamin D_3_ hydroxyderivatives on VDR translocation from cytoplasm to nucleus (Figure 10). As shown in Figure 10, there is a dose-dependent ligand-induced translocation of VDR to the nucleus, with a similar effect for both 20(OH)D_3_ (100 nM) and 1,25(OH)_2_D_3_ at a concentration of 100 nM (Figure 10, insert). These results are consistent with the previously reported effect for 20-hydroxyvitamin D2 effect (Slominski *et al*, 2011).

## Discussion

Malignant melanoma is a highly aggressive form of skin cancer that is extremely difficult to treat because it is highly resistant to conventional chemotherapeutic agents and radiation treatment (Soengas and Lowe, 2003). Nuclear factor-*κ*B is constitutively activated in malignant melanoma and other cancers, and is believed to confer resistance to cytotoxic therapies by suppressing the induction of apoptosis (Franco *et al*, 2001; Wang and Richmond, 2001; Yang and Richmond, 2001; Karin *et al*, 2002). Many mechanisms are responsible for the elevated level of NF-*κ*B activity in malignant melanoma (Lopez-Bergami *et al*, 2008). In melanoma cells, the NF-*κ*B pathway can be altered by upregulation of the p50 and p65 NF-*κ*B subunits (Meyskens *et al*, 1999; McNulty *et al*, 2001) and downregulation of the NF-*κ*B inhibitor, I*κ*B (Yang and Richmond, 2001; Dhawan and Richmond, 2002). The ability to synthesise melanin is an example of differentiation programme in normal and malignant melanocytes with usually decreased proliferative potential (Slominski *et al*, 2004). Using a human melanoma line in which melanin pigmentation can be induced by media with increased concentration of melanin precursors (Slominski *et al*, 2009), we demonstrate that pigmentation affects NF-*κ*B activity (Figure 1). The p65/p50 heterodimers of NF-*κ*B are the predominant complex observed in activated cells and play an important role in regulating gene transcription after nuclear translocation. As determined by various assays, we found high nuclear levels of the p65 NF-*κ*B subunit in nonpigmented cells, whereas p65 was localised in the cytoplasm of pigmented cells.

The high constitutive activity of NF-*κ*B in nonpigmented melanoma cells is consistent with a role for NF-κB in the malignant characteristics of human melanoma (Yang and Richmond, 2001). Therefore, we next examined the status of NF-*κ*B activation by immunohistochemical analysis of archival clinical material from 79 cases of melanomas. Consistent with our observations from the cell culture model of inducible melanogenesis, a predominant nuclear location for p65 was observed in specimens comprising amelanotic cells, whereas predominantly cytoplasmic location of p65 was observed in specimens containing melanotic cells. Furthermore, the translocation of NF*κ*B into the cell nuclei was accompanied by higher proliferation activity as assessed by Ki67 immunostaining and mitotic index.

Melanin pigment not only protects melanocytes against the harmful effects of solar radiation (Slominski *et al*, 2004; Lin and Fisher, 2007), but can also increase the resistance of melanoma cells to various forms of anticancer therapy (Meyskens *et al*, 2007; Brozyna *et al*, 2008; Slominski *et al*, 2009). Vitamin D, an important nutritional factor, protects against melanomagenesis (Berwick *et al*, 2005; Egan, 2009; Newton-Bishop *et al*, 2009). In addition, 20(OH)D_3_, a novel product of vitamin D3 metabolism by CYP11A1 (Slominski *et al*, 2005), has strong antiproliferative activity in keratinocytes (Zbytek *et al*, 2008) and anticancer activity in leukaemia lines (Slominski *et al*, 2010). The biological activities of novel 20(OH)D_3_ and classical 1,25(OH)_2_D_3_ are mediated by binding to the VDR (Deeb *et al*, 2007; Zbytek *et al*, 2008), which is ubiquitously expressed in cells. Therefore, we compared the sensitivity of nonpigmented and pigmented melanoma cells with these forms of vitamin D. First, we have shown that treatment with vitamin D3 derivatives causes VDR translocation to the nucleus. Second, both 20(OH)D_3_ and 1,25(OH)_2_D_3_ exhibited a concentration-dependent inhibition of melanoma cell proliferation, with nonpigmented cells being more sensitive to 20(OH)D_3_ and 1,25(OH)_2_D_3_ treatment than pigmented cells. The most logical explanation for these differences is reduced VDR expression in pigmented cells compared with nonpigmented cells (see Figure 1), a finding further confirmed by analysis of clinical material (see Figure 8).

The NF-*κ*B pathway is a promising anticancer target. In melanoma, gene transfer approaches have been used to inhibit the NF-*κ*B pathway by inactivating RelA (McNulty *et al*, 2001) or by overexpressing I*κ*B (Huang *et al*, 2000). Interestingly, we demonstrated that NF-*κ*B activity was inhibited by 1,25(OH)_2_D_3_ (Harant *et al*, 1997) and 20(OH)D_3_ (Janjetovic *et al*, 2009). The latter effect was accompanied by decreased keratinocyte cell proliferation (Zbytek *et al*, 2008). In this study, using melanoma models, we found that 20(OH)D_3_ selectively inhibited NF-*κ*B activity in nonpigmented human melanoma cells, having little effect on melanised cells. A similar effect was observed with the classical vitamin D derivative 1,25(OH)_2_D_3_. Accordingly, this places a limitation on the use of this pathway in adjuvant treatment of pigmented melanomas. However, treatment of nonpigmented cells with the secosteroids (1,25(OH)_2_D_3_ and 20(OH)D_3_) resulted in a time-dependent inhibition of NF-*κ*B activity that correlated with suppression of NF-*κ*B activation, as measured by ELISA and NF-*κ*B-dependent transcription reporter assays. Furthermore, secosteroid treatment of nonpigmented cells blocked the constitutive nuclear translocation of p65 subunit of NF-*κ*B and resulted in p65 retention in the cytoplasm. Thus, downregulation of NF-*κ*B represents a novel approach to treat amelanotic or poorly pigmented melanomas. In this context, application of nutritional factors (vitamin D) or its nontoxic 20(OH)D_3_ derivative, which can be classified as a natural product (it is generated by an action of an enzyme or by isolated mitochondria; Slominski *et al*, 2005), can represent a new adjuvant therapy. This approach is also justified by recent clinical and epidemiological studies in melanoma, showing vitamin D levels to be associated with lower melanoma incidence (Egan, 2009; Newton-Bishop *et al*, 2009; Pinczewski and Slominski, 2010), and histopathological analyses showing that progression of melanoma is linked to reduction of VDR expression and lack of VDR expression is accompanied by shorter overall survival time (Brozyna *et al*, 2011).

In summary, this study highlights the importance of the NF-*κ*B pathway in the progression and growth of melanoma cells. Our data indicate that this pathway is a potential target for the treatment of malignant melanoma with vitamin D3 prohormone or its active hydroxyderivatives. However, the final outcome will depend on the pigmentary phenotype. Importantly, the noncalcemic vitamin D3 derivative, 20(OH)D_3_, can target NF-*κ*B and regulate melanoma progression. We also show that the high melanin content in melanomas is associated with melanoma resistance to treatment by vitamin D3 derivatives.

## Figures and Tables

**Table 1:** NF-*κ*B nuclear localisation in melanomas

	**NF-*κ*B nuclear localisation**					
	**Mean** a	**a** b	**b** b	**c** b	**d** b	**e** b
Amelanotic melanomas	39.7±24.4	14.8%	33%	33%	18.5%	11%
Moderately pigmented melanomas	22.5±19.2	37.5%	31%	21.8%	6%	3%
Strongly pigmented melanomas	10.8±5.0	80%	15.8%	5%	0%	0%

Abbreviation: NF-*κ*B=nuclear factor-*κ*B.

a Data presented as mean value±s.d. of percentage of cells with nuclear NF-*κ*B localisation.

b Data presented as number of cases (%) with NF-*κ*B localised in nuclei/number of total cases: a (0–10%), b (10–25%), c (25–50%), d (50–75%), and e (75–100%).
