# Supplementary figures and images for: High basal NF-κB activity in nonpigmented melanoma cells is associated with an enhanced sensitivity to vitamin D3 derivatives
Source: Br J Cancer. 2011 Nov 17;105(12):1874–84. doi: 10.1038/bjc.2011.458 (PMC3251881; doi:10.1038/bjc.2011.458)

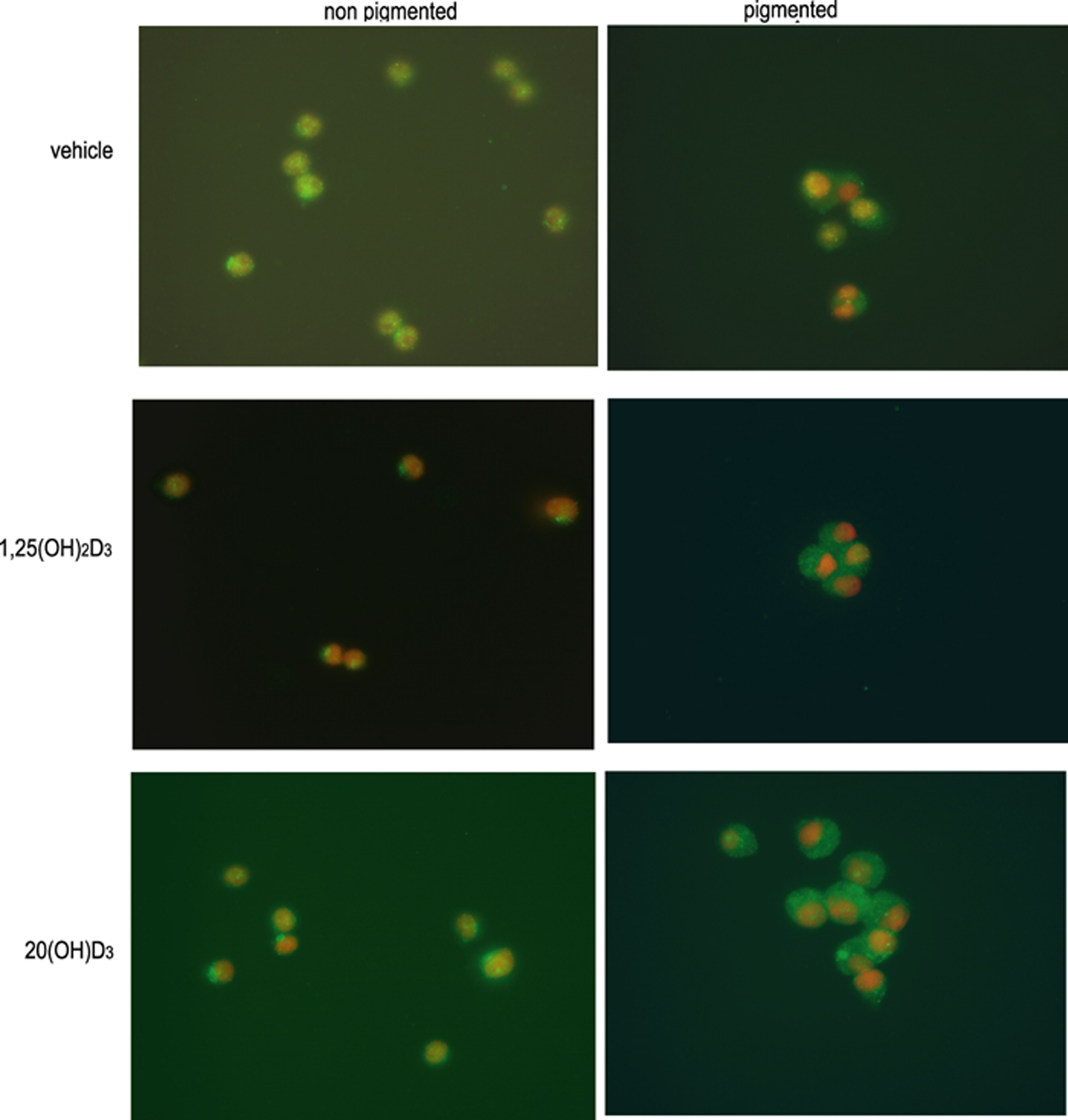

Supplement: Supplementary Figure 1 [file bjc2011458x1.tif]
