# Supplementary material for: High basal NF-κB activity in nonpigmented melanoma cells is associated with an enhanced sensitivity to vitamin D3 derivatives
Source: Br J Cancer. 2011 Nov 17;105(12):1874–84. doi: 10.1038/bjc.2011.458 (PMC3251881; doi:10.1038/bjc.2011.458)
Supplement: Supplementary Figure Legend [file bjc2011458x2.doc]

**Supplemental Figure 1**. Immunofluorescent staining for p65 in melanoma cells after treatment with 20(OH)D3 or 1,25(OH)_2_D3.

Human SKMEL-188 nonpigmented and pigmented melanoma cells were incubated for 1 h with 100 nM 20(OH)D_3_ or 1,25(OH)_2_D_3_, or vehicle (ethanol), and then fixed and incubated with primary antibody against p65, followed by secondary antibody linked to FITC. Nuclei were stained red with PI (magnification 40X).
